# Study of the Adsorption and Separation Behavior of Scandium and Zirconium by Trialkyl Phosphine Oxide-Modified Resins in Sulfuric and Hydrochloric Acid Media

**DOI:** 10.3390/toxics12050350

**Published:** 2024-05-10

**Authors:** Botao Xu, Xiangbiao Yin, Shunyan Ning, Yilai Zhong, Xinpeng Wang, Toyohisa Fujita, Mohammed F. Hamza, Yuezhou Wei

**Affiliations:** 1 State Key Laboratory of Featured Metal Materials and Life-Cycle Safety for Composite Structures, School of Resources, Environment and Materials, Guangxi University, Nanning 530004, China; 2115391075@st.gxu.edu.cn (B.X.); 2015391073@st.gxu.edu.cn (Y.Z.); wangxinpeng@gxu.edu.cn (X.W.); fujitatoyohisa@gxu.edu.cn (T.F.); 2 School of Nuclear Science and Technology, University of South China, 28 Changsheng West Road, Hengyang 421001, China; ningshunyan@usc.edu.cn (S.N.); m_fouda21@usc.edu.cn (M.F.H.); 3 School of Nuclear Science and Engineering, Shanghai Jiao Tong University, 800 Dong Chuan Road, Shanghai 200240, China

**Keywords:** TRPO/SiO_2_-P, scandium, zirconium, adsorption, separation

## Abstract

Zirconium is recognized as one of the main impurities of the rare earth element scandium during purification. It presents significant challenges due to its similar chemical properties, making separating it difficult. This study used trialkyl phosphine oxide (TRPO) as a functional ligand, and the effects of carrier type and acidity on adsorption performance were first investigated. Among these, the novel extraction resin SiO_2_-P as a carrier for TRPO demonstrated more prominent separation performance in 0.2 M H_2_SO_4_ and 5 M HCl solutions. The kinetic and isotherm data were consistent with the pseudo-secondary kinetics and Langmuir model, respectively, and the adsorption process could be regarded as homogeneous monolayer adsorption subject to the dual effects of chemisorption and internal diffusion. In addition, thermodynamic analysis showed that the adsorption process of zirconium under the experimental conditions was a spontaneous endothermic process. Combined with the results of SEM-EDS, FT-IR, and XPS analyses, scandium and zirconium were successfully adsorbed by the resin and uniformly distributed on its surface, and the greater affinity of the P=O groups on the resin for zirconium was the critical factor contributing to the separation of scandium and zirconium. Finally, scandium and zirconium in sulfuric acid and hydrochloric acid media were extracted and separated by column experiments, and the purity of scandium could reach 99.8% and 99.99%, respectively.

## 1. Introduction

Due to their similar chemical behavior, scandium, yttrium, and the 15 lanthanide elements constitute rare earth elements [1]. Occupying the 21st position on the periodic table, scandium is regarded as a critical metal for future applications [2]. Recently, scandium has been utilized as a material for aero-engines and spacecraft, owing to its excellent resistance to high temperatures and corrosion, along with its high strength and resistance to deformation [3,4,5]. Furthermore, the properties of scandium enabled it to play a crucial role in various fields, including catalytic materials [6,7], electronic information [8,9], luminescent materials [10], and hydrogen storage materials [11,12]. Despite scandium’s wide range of applications, its availability is limited. This limitation stems from its complex deposit forms and difficulty in extraction, despite being the 31st most abundant element in the earth’s crust [13]. A 2019 survey [9] revealed that the price of scandium oxide reaches a staggering USD3800/kg, significantly limiting its applications. Currently, scandium is primarily recovered as a by-product from sources including tungsten refining slag, uranium leach solution, titanium pigment production waste, and red mud [14]. Prevalent recovery methods include solvent extraction [15,16,17], ion exchange [18,19,20], and adsorption [21,22,23]. Solvent extraction, vital for analyzing and preparing high-purity substances and widely used in hydrometallurgy, faces challenges such as co-extraction of impurities, saponification, low selectivity, and low dissolution rates [24]. Ion exchange methods tend to be less selective for target elements. Conversely, the adsorption method offers advantages such as simplicity, no secondary pollution, strong ion selectivity, and high adsorption efficiency. Hence, it is a promising technique for recovering and purifying scandium.

**Figure 1:**
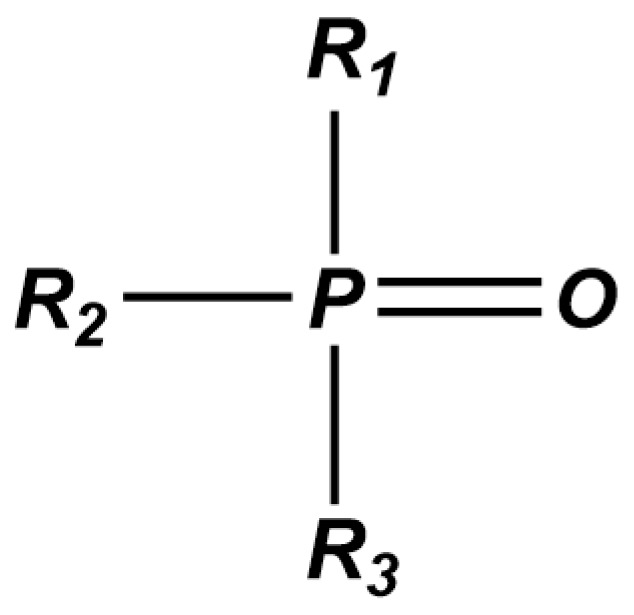
Chemical structure of TRPO (R_1_, R_2_, R_3_: any of the three, i.e., hexyl, heptyl, or octyl group).

Metallic zirconium is mainly used in the manufacture of materials such as nuclear fuel cladding, chemical piping, and heat exchangers due to its advantages such as low thermal neutron cross-section, high melting point, machinability, and corrosion resistance [25,26]. Zirconium frequently co-occurs with scandium in its purification process, complicating the accurate separation of these elements due to their similar chemical properties [27]. Zirconium oxide is the primary impurity in market-available scandium oxide products. The significant price disparity between scandium oxide of varying purities can be attributed to the challenges in preparation and the varying application values of these materials. As an illustration, yttrium-scandium-gallium garnet (YSGG) was produced by adding Sc_2_O_3_ of 99.9–99.99% purity to yttrium-gallium garnet (YGG), and the emission power of the latter was three times higher than that of the former [28], while Joseph et al. [29] discovered that the purity of the scandium source in the heterostructures of Sc_x_Al_1-x_N/GaN significantly altered the chemical and electronic properties of the heterostructures; in the case of Sc_x_Al_1-x_N/GaN with a purity of 99.9%~99.99% Sc_x_Al_1-x_N/GaN, the unintentional doping density of carbon, oxygen, and fluorine in the latter decreased by 2–3 orders of magnitude, and the leakage of the grown film decreased by 5–7 orders of magnitude. Consequently, the cost-effective production of high-purity scandium oxide emerges as a critical factor for commercial competitiveness. Various methods have been explored for removing impurity zirconium during scandium purification. For instance, Zhang et al. [30] developed a functionalized silica-based resin through an in situ polymerization process, with dynamic adsorption experiments in 0.5 M HNO_3_ and HCl achieving Sc(III) purities of 99.97% and 99.94%, respectively. Similarly, Chen et al. [21] impregnated the extractant TODGA into silica-based composites under vacuum, yielding adsorbents demonstrating a Zr removal rate of over 99% in separation experiments with crude Sc_2_O_3_ products. Moreover, trialkyl phosphine oxide (TRPO, the chemical structure shown in Figure 1) has proven effective as an extractant for scandium recovery and purification in solvent extraction processes involving titanium dioxide waste acid [31].

This study discussed the separation characteristics of TRPO-modified silica-based resins for scandium and zirconium within sulfuric and hydrochloric acid media. Utilizing TRPO as a functional ligand, the carrier (SiO_2_-P) comprised porous silica microspheres with an inner surface modified by styrene-divinylbenzene copolymer, while the TRPO/SiO_2_-P resin was created through vacuum impregnation. In contrast to traditional polymer-based resins, which have particle sizes of 0.3–3 mm, this novel extraction resin features a smaller size (75–150 μm). It boasts fast kinetics, high strength, and good column mobility [32]. Initially, this study examined the adsorption of scandium and zirconium by TRPO on various polymer-based resins, followed by an evaluation of the resins’ performance for separating scandium and zirconium in sulfuric and hydrochloric acid media via intermittent adsorption experiments. This study employed SEM-EDS, FT-IR, and XPS analysis to elucidate the adsorption mechanism. Ultimately, the research explored the separation performance of TRPO/SiO_2_-P for scandium and zirconium through a dynamic column, suggesting a novel approach for removing zirconium impurities from high-purity Sc_2_O_3_.

## 2. Experimental

### 2.1. Materials

Scandium oxide (Sc_2_O_3_) (purity > 99%) was provided by Guangxi Scandium New Energy Technology Co, Ltd. (Wuzhou, China) Scandium sulfate octahydrate (Sc_2_(SO_4_)_3_·8H_2_O), zirconium sulfate tetrahydrate, (Zr(SO_4_)_2_·4H_2_O), scandium chloride hexahydrate (ScCl_3_·6H_2_O), and zirconium oxychloride octahydrate (ZrOCl_2_·8H_2_O) were purchased from Shanghai Macklin Biochemical Co., Ltd. (Shanghai, China) Oxalic acid dihydrate (H_2_C_2_O_4_·2H_2_O) was purchased from Sinopharm Chemical Reagent Co., Ltd. (Shanghai, China) Scandium and zirconium standard solutions (1000 ppm) were provided by the National Center for Analysis and Testing of Nonferrous Metals and Electronic Materials. TRPO (purity > 93%) and sulfonated kerosene (industrial purity for use as a diluent) were purchased from Lai Yashi Chemical Co., Ltd., Shanghai, China. XAD7HP was purchased from Shanghai Macklin Biochemical Co., Ltd. (Shanghai, China), HZ-635 was provided by Shanghai East China University of Science and Technology, and the chemical structures of these two materials are shown in Figure S1. SiO_2_-P was produced by our laboratory, and all other reagents were analytically pure. XAD7HP is a methacrylic acid polymer with a diameter of about 300–900 μm, a specific surface area of 507 m^2^/g, and a pore size of 11 nm. HZ-635 is a resin sphere with a diameter of about 250 μm, specific surface area, and pore size of 730 m^2^/g and 8 nm, respectively, and is a styrene-divinylbenzene polymer. As for SiO_2_-P, silica spheres were used as the skeleton in which styrene and divinylbenzene were polymerized in situ to obtain the organic–inorganic carrier SiO_2_-P, which had an average diameter of about 75~150 μm, and pore size and specific surface area of about 98 m^2^/g and 34 nm, respectively [33].

### 2.2. Synthesis of TRPO/SiO_2_-P Adsorbent

In our previous work [32,34], we have synthesized a batch of novel extractive resin microspheres (SiO_2_-P) with a pore fraction of 0.69 and a diameter of 40–60 μm, where “P” refers to an inert styrene-divinylbenzene copolymer, which is simply styrene and divinylbenzene embedded in the pores of silica by in situ polymerization. Briefly, styrene and divinylbenzene were embedded into the silica pores by in situ polymerization, and SiO_2_-P with a polymer content of 17–18 w% was finally obtained. The synthesis step of TRPO/SiO_2_-P was referred to as the method in the literature [35,36]. Specifically, CH_2_Cl_2_ was used as the solvent, TRPO was fully dissolved, and then a certain mass of SiO_2_-P was added (mTRPO:nSiO_2_-P = 1:2). The mixture was subjected to decompression distillation in a cigar-shaped flask. The solvent carrying TRPO was pressed under negative pressure. Under negative pressure, the solvent carrying TRPO will be pressed into the pores of SiO2-P, and the solvent will be evaporated until all of it is evaporated under decreasing air pressure. Finally, TRPO will be left in the pores to obtain TRPO/SiO_2_-P.

### 2.3. Characterization

SEM-EDS analysis was performed using a HITACHI SU8200 equipped with a Pro-X unit to characterize the micro-morphology and chemical composition distribution of the TRPO/SiO_2_-P resin before and after adsorption. The structural and group information of the resin and the changes in chemical bonding before and after adsorption were analyzed by Fourier transform infrared spectroscopy (Shimadzu, IRTracer-100, Kyoto, Japan). The samples were analyzed by X-ray photoelectron spectroscopy (XPS) using an ESCALAB 250XI device and the valence of the elemental composition of the material was measured using a monochromatic Al-Ka radiation source to determine the changes in the elemental valence of the resin before and after adsorption and in the chemical environment.

### 2.4. Batch Adsorption Experiment

In order to investigate the adsorption behavior of the adsorbent on scandium and zirconium in sulfuric and hydrochloric acid media, we used different carriers and acidity, scandium–zirconium molar ratios, scandium–zirconium initial concentrations, and solid–liquid ratios as the variables to explore the effects of the adsorption effects, respectively. The adsorption capacity q, adsorption efficiency E, desorption capacity $q_{d}$, desorption efficiency $E_{d}$, distribution coefficient $K_{d}$, and separation factor ${SF}_{Zr/Sc}$ were used to represent the adsorption performance of the adsorbent, respectively. The specific formulas were calculated as follows:

(1) $$q=\frac{{(C}_{0}-C)}{m}\times V$$

(2) $$E=\frac{{(C}_{0}-C)}{C_{0}}\times 100\%$$

(3) $$q_{d}=C_{d}\times \frac{V}{m}$$

(4) $$E_{d}=\frac{q_{d}}{q}\times 100\%$$

(5) $$K_{d}=\frac{{(C}_{0}-C)}{C}\times \frac{V}{m}$$

(6) $${SF}_{A/B}=K_{d_{A}}/K_{d_{B}}$$

where $C_{0}$, C, and $C_{d}$ denote the metal ion concentration in the aqueous solution before, after adsorption, and after desorption, respectively. V and m denote the volume of the liquid phase during adsorption and the mass of the TRPO/SiO_2_-P resin in a completely dry state, respectively.

### 2.5. Column Separation Experiment

The column experiment (with dimension 0.5 cm × 10 cm) was used for simulating the separation behavior of scandium and zirconium in applying the absorbent. A specific mass of dried resin was filled into the column by the wet-filling method, and the peristaltic pump (EYELA, MP2000) was adjusted to keep the flow rate of the feed solution at 0.2 mL·min^−1^; finally, the effluent was collected by a fraction collector. A sketch of the column system is shown in Figure S2. During this process, the concentration of metal ions in the outlet solution was measured by ICP-AES.

## 3. Results and Discussion

### 3.1. Batch Adsorption Experiment

#### 3.1.1. Effect of Carrier and Acidity

**Figure 2:**
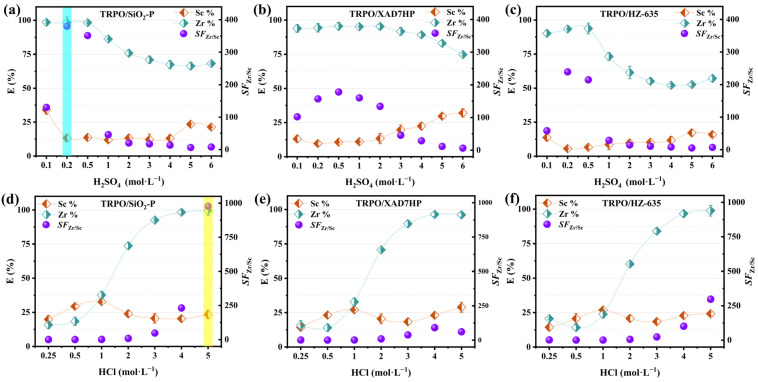
Effect of different carriers and acidity on the adsorption performance (**a**,**d**) TRPO/SiO_2_-P, (**b**,**e**) TRPO/XAD7HP, (**c**,**f**) TRPO/HZ-635 (m/V = 0.1 g/5 mL, Sc and Zr each 5 mM (mixture), T = 298 K, shaking speed: 140 rpm, t = 24 h).

TRPO was impregnated into three different carriers for the adsorption of scandium and zirconium ions to evaluate the effect of carrier type on resin performance. In order to determine the optimal acidic environment for adsorbent efficacy, a series of experiments with different pH values were conducted, along with an assessment of carrier factors, the results of which are shown in Figure 2. For comparison, the corresponding SF values of the three resins at the optimal acidity were organized in Table 1. It can be seen that TRPO/SiO_2_-P exhibited superior separation performance in sulfuric acid (0.2 M) and hydrochloric acid (5 M) media, with separation factors (SF) of 380 and 977, respectively, when compared to TRPO/XAD7HP and TRPO/HZ-635. The adsorption kinetics of the three materials were also investigated (Figure S3), and overall, TRPO/SiO_2_-P adsorbed zirconium faster and in larger quantities with little scandium adsorption. In conclusion, the novel extraction process of TRPO with SiO_2_-P as a carrier in sulfuric and hydrochloric acid media can separate scandium and zirconium more efficiently.

#### 3.1.2. Effect of Sc/Zr Ratio and V/m Ratio

**Figure 3:**
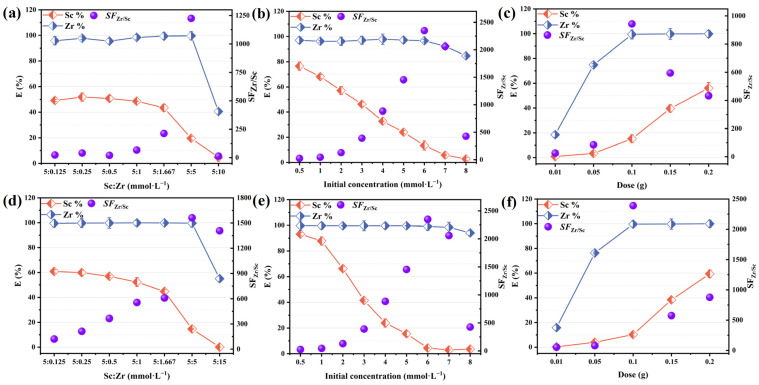
Effect of (**a**) Sc/Zr molar ratio, (**b**) initial concentration of Sc and Zr, and (**c**) solid−liquid ratio on resin properties in 0.2 M H_2_SO_4_ solution. Effect of (**d**) Sc/Zr molar ratio, (**e**) initial concentration of Sc and Zr, and (**f**) solid−liquid ratio on resin properties in 5 M HCl solution ((**a**,**b**,**d**,**e**) m/V = 0.1 g:5 mL, (**c**,**f**) Sc and Zr each 5 mM (mixture), V = 5 mL; T = 298 K, shaking speed: 140 rpm, t = 24 h).

In this study, the effect of scandium–zirconium molar ratio on the separation performance of TRPO/SiO_2_-P was investigated, i.e., keeping the concentration of Sc constant and increasing the concentration of zirconium, and the results obtained are shown in Figure 3a,d. A noteworthy phenomenon is that as the scandium–zirconium molar ratio decreases (i.e., from 40 to 1), the separation performance of the adsorbent gradually improves (Figure 3a: SF value increases from 24 to 1225; Figure 3d: SF value increases from 121 to 1562). This is because at low zirconium ion concentrations, after the adsorbent adsorbs all the zirconium ions in solution, many internal adsorption sites remain vacant, resulting in partial adsorption of scandium ions and insignificant separation performance. As the concentration of zirconium ions increased from 0.125 M to 5 M, the vacant adsorption sites in the adsorbent were occupied by more zirconium ions. At the same time, a decrease in the scandium adsorption rate was observed, which was hypothesized to be the result of zirconium ions replacing some of the sites occupied by scandium, thus improving the separation performance of the adsorbent. The improvement may be attributed to the fact that the adsorbent has a greater affinity for zirconium ions than for scandium ions. The separation factor, on the other hand, decreased dramatically when the concentration of zirconium ions was increased to 15 M. This was because the adsorbent was saturated, resulting in some of the remaining zirconium ions in the solution not being adsorbed. Interestingly, the adsorbent maintains separation efficacy even at a scandium to zirconium molar ratio of 40:1 (SF > 10), which suggests that it has promising value for practical applications.

Figure 3b,e shows the performance of separation influenced by the initial concentration, with the Sc/Zr molar ratio maintained at 1:1. The outcomes aligned with those observed in the molar ratio experiments. Figure 3c,f reports the impact of the solid–liquid ratio on adsorption performance, and it was observed that for 100% removal of zirconium ions, the mass of the adsorbent reached 0.1 g. Subsequent increases in adsorbent mass paradoxically diminished the separation performance, as the quantity of zirconium ions was insufficient to occupy the gradually increasing adsorption sites that were used for scandium ions. In summary, the adsorption of scandium and zirconium ions by the adsorbent exhibited a clear order for preference of Zr(IV) over Sc(III).

#### 3.1.3. Kinetic Analysis

**Figure 4:**
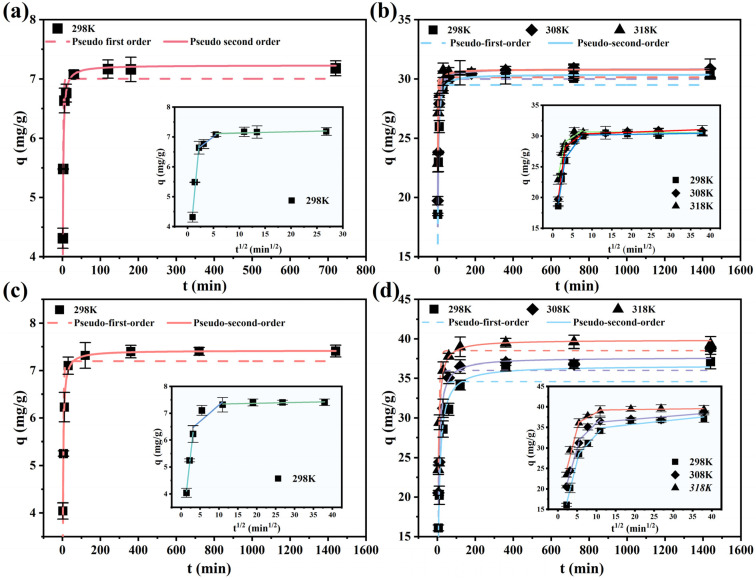
Adsorption kinetics of TRPO/SiO_2_-P resin towards (**a**) Sc and (**b**) Zr in 0.2 M H_2_SO_4_ solution and (**c**) Sc and (**d**) Zr in 5 M HCl solution (m/V = 0.1 g/5 mL, Sc or Zr concentration = 8 mM, T = 298 K, shaking speed: 140 rpm).

Further investigation was conducted on the adsorption behavior of scandium and zirconium ions by TRPO/SiO_2_-P in isolated sulfuric and hydrochloric acid media. Figure 4a,b indicates that in sulfuric acid media, the adsorption equilibrium for Sc(III) and Zr(IV) was reached after 2 h and 1 h, respectively, on the other hand, it was achieved after 2 and 6 h, respectively, in the hydrochloric acid media (Figure 4c,d). The experimental data from Figure 4 were analyzed using pseudo-first-order (Equation (S1)) and pseudo-second-order (Equation (S2)) kinetic models, with the fitting parameters detailed in Table 2 and Table 3. The correlation coefficients from these data (R_2_^2^ > R_1_^2^) indicate that the experimental data align more closely with the pseudo-second-order kinetic model. This implies that the adsorption processes for both Sc(III) and Zr(IV) are predominantly governed by chemisorption [37].

To ascertain the controlling steps of the adsorption process, adsorption data were analyzed using the Weber−Morris internal diffusion (Equation (S3)) model; Figure 4 shows kinetic results, which are displayed as insets in individual plots. Tables S1 and S2 organize the fitted parameters, where the magnitude of the k-value indicates the relative speed of the adsorption rate. Evidently, the adsorption process for Sc(III) and Zr(IV) by the adsorbent comprises three stages. The initial stage exhibits the fastest adsorption rate, attributable to the abundance of internal adsorption sites available for bonding with Sc(III) and Zr(IV) upon initial contact. The reduced rate in the second stage is a consequence of the decreasing number of available adsorption sites within the adsorbent. In the third stage, the k-value approaches zero, signifying that the adsorbed amount stabilizes and the adsorption process reaches equilibrium [38]. Given that none of the fitted curves in the insets pass through the coordinate system’s origin (0, 0), this suggests that the adsorption process involves not just chemisorption but also internal diffusion processes [39].

#### 3.1.4. Adsorption Isothermal

**Figure 5:**
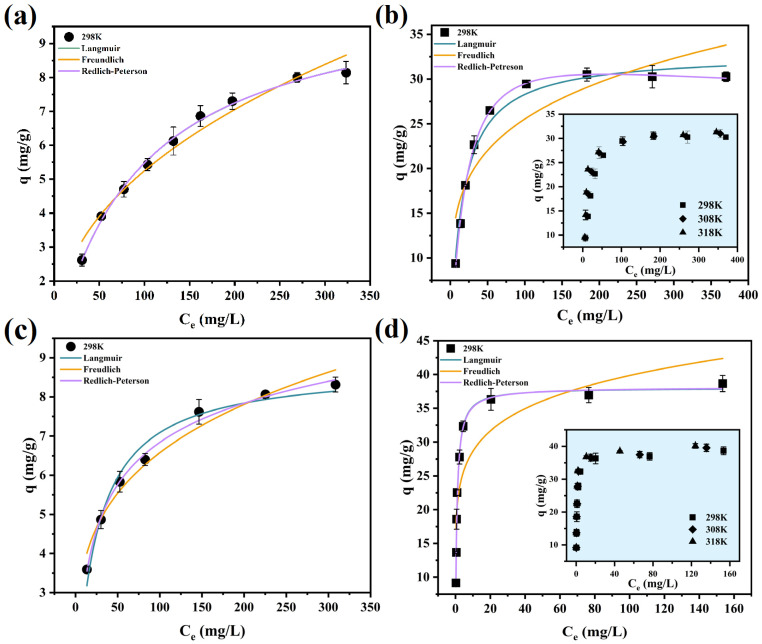
Adsorption isothermal of TRPO/SiO_2_-P resin towards (**a**) Sc and (**b**) Zr in 0.2 M H_2_SO_4_ solution and (**c**) Sc and (**d**) Zr in 5 M HCl solution (m/V = 0.1 g/5 mL, T = 298K, shaking speed: 140 rpm, t = 12 h).

Isotherm data for the adsorbent in both acidic media are presented in Figure 5. The data were analyzed using Langmuir (Equation (S4)), Freundlich (Equation (S5)), and Redlich–Peterson (Equation (S6)) isothermal models, with the corresponding results detailed in Table 4 and Table 5. The Langmuir model posits that the adsorption process constitutes homogeneous monolayer adsorption, occurring exclusively at a finite number of identical and equivalent sites [40]. Typically, the Freundlich model is associated with non-homogeneous multilayer adsorption. This model suggests that the adsorbent’s maximum adsorption capacity is cumulative adsorption at all sites, with the most potent adsorption sites being occupied initially [41]. Within the Freundlich model parameters, a 1/n value between 0 and 1 indicates a chemisorption process, whereas a 1/n value greater than 1 implies synergistic adsorption involving both physical and chemical interactions [42]. The Redlich–Peterson isothermal model, a three-parameter construct, simplifies to a linear isothermal model at low surface coverage. It aligns with the Langmuir model when the parameter g equals 1 [43,44].

A comparison of the data in Table 4 and Table 5 indicates that both Langmuir and Redlich–Peterson models provide a superior fit. Notably, the g-value in the Redlich–Peterson model parameters is very close to 1. Consequently, it is reasonable to infer that the adsorption process adheres to the Langmuir model, characterized by homogeneous monolayer adsorption. According to the Langmuir model fitting results in the table, the maximum adsorption capacities (q_m_) of the adsorbent for Sc(III) and Zr(IV) in sulfuric and hydrochloric acids at 298 K were determined to be 10.71 mg·g^−1^, 8.77 mg·g^−1^, 32.83 mg·g^−1^, and 38.03 mg·g^−1^, respectively.

#### 3.1.5. Thermodynamic Analysis

Considering that the adsorbent has a stronger affinity for zirconium ions, the effect of temperature on the adsorption process of zirconium ions was investigated separately, and the results are shown in Figure 5b,d. The thermodynamic parameters of the adsorption process follow Equations (6) and (7):

(7) $$lnK_{L}=-\frac{∆H^{0}}{RT}+\frac{{∆S}^{0}}{R}$$

(8) $${∆G}^{0}={∆H}^{0}-T{∆S}^{0}$$

where ${∆G}^{0},{∆H}^{0}$, and ${∆S}^{0}$ are the changes in standard Gibbs free energy (J·mol^−1^), standard enthalpy (J·mol^−1^), and standard entropy (J·mol^−1^·K^−1^), respectively, R is the universal constant (8.314 J·mol^−1^·K^−1^), and $K_{L}$(L·mol^−1^) is the Langmuir constant at temperature T(K).

**Figure 6:**
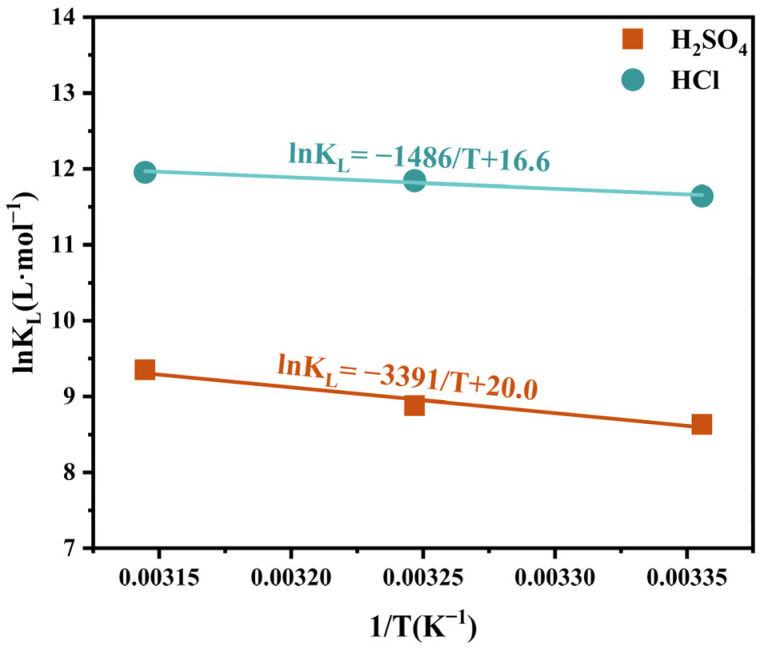
Plot of $lnK_{L}$ versus 1/T.

A linear relationship between $lnK_{L}$ and 1/T has been plotted (Figure 6); the determination of parameters ${∆H}^{0}$ and ${∆S}^{0}$ values from the slopes and intercepts, respectively. The relevant parameters are summarized in Table 6. As indicated by the results in Table 6, positive ${∆H}^{0}$ values suggest that the adsorption of Zr(IV) by the adsorbent in sulfuric and hydrochloric acid media is an endothermic process. The positive ${∆S}^{0}$ values imply a decrease in surface ordering during the adsorption of zirconium ions by the adsorbent. A negative ${∆G}^{0}$ value denotes the spontaneous nature of zirconium ion adsorption by the adsorbent [45]. According to Table 4 and Table 5, the adsorption capacity of the adsorbent for zirconium exhibited a slight increase (approximately 1~2 mg·g^−1^) with rising temperatures. Given that zirconium adsorption is a heat-absorbing process, it can be inferred that elevated temperatures marginally enhance the adsorption of zirconium ions, although the effect is relatively modest.

### 3.2. Adsorption Mechanism

#### 3.2.1. SEM-EDS Analysis

**Figure 7:**
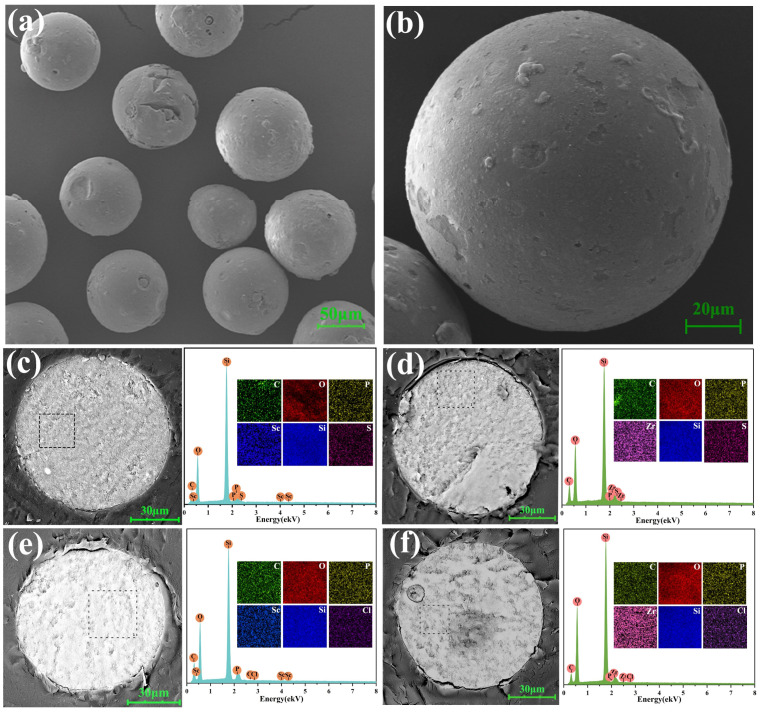
SEM-EDS of Bulk (**a**) and individual (**b**) fresh TRPO/SiO_2_-P, TRPO/SiO_2_-P-Sc (**c**), and TRPO/SiO_2_-P-Zr (**d**) in H_2_SO_4_ medium and TRPO/SiO_2_-P-Sc (**e**) and TRPO/SiO_2_-P-Zr (**f**) in HCl medium.

The micrograph of TRPO/SiO_2_-P was shown in Figure 7a,b, which showed that the TRPO/SiO_2_-P resin is a spherical structure composed of particles, each with a relatively uniform diameter of about 100 µm. EDS results investigated the elemental composition of the resin after adsorption. The results showed that carbon (C), nitrogen (N), sulfur (S), chlorine (Cl), oxygen (O), scandium (Sc), and zirconium (Zr) were uniformly distributed in the cross-section of the adsorption-treated TRPO/SiO_2_-P (Figure 7c–f). This indicates the successful adsorption of scandium and zirconium by the resin, and the presence of sulfur and chlorine elements in the cross-section suggests that acidic radical ions (SO_4_^2−^ and Cl^−^) are also involved in the adsorption process.

#### 3.2.2. FT-TR Analysis

**Figure 8:**
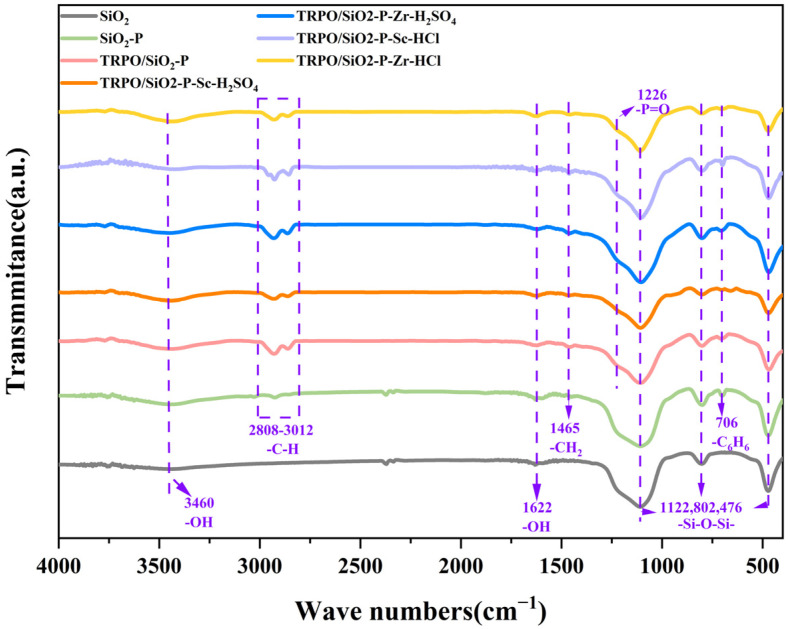
FT-IR of SiO_2_, SiO_2_-P, and TRPO/SiO_2_-P before and after adsorption of Sc(III) and Zr(IV) in sulfuric and hydrochloric acid media.

In Figure 8, all samples exhibited weak adsorption bands at 1622 and 3460 cm^−1^, which is attributable to the bending vibrations of hydroxyl groups in the adsorbed water on the samples [46]. Bands at 2808–3012 cm^−1^ correspond to the stretching vibrational peak of –C–H aliphatic [47]. Notably, the peaks of C–H for TRPO/SiO_2_-P are more pronounced than that for SiO_2_-P, suggesting that the presence of TRPO increases the –C–H content. An absorption band for the benzene ring is evident at 706 cm^−1^, and the band at 1465 cm^−1^ results from the stretching vibration of –CH_2_ [47,48]. The absorption bands appearing at 476, 802 cm^−1^ are asymmetric stretching vibrations of the –Si–O–Si– bonds, while the absorption band at 1122 cm^−1^ is attributed to the symmetric stretching vibration of the same group [49]. At 1226 cm^−1^, the bending vibrational peak of the –P=O bond is present; however, changes in the –P=O bond’s appearance before and after adsorption are obscured by the influence of the –Si–O–Si– peak [36].

#### 3.2.3. XPS Analysis

**Figure 9:**
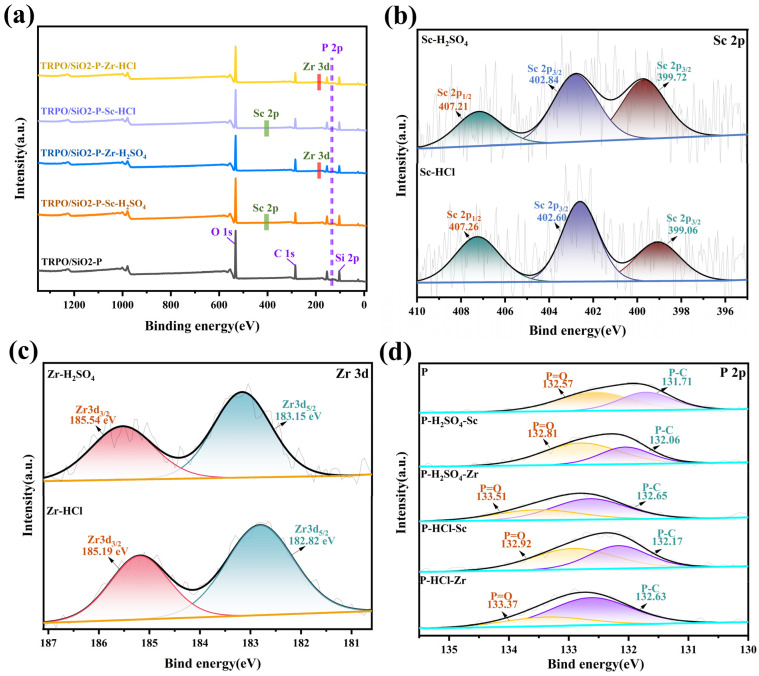
(**a**) XPS full spectra of TRPO/SiO_2_-P, (**b**) high-resolution Sc 2p spectra, (**c**) high-resolution Zr 3d spectra, and (**d**) high-resolution P 2p spectra.

The mechanism of interaction between TRPO/SiO_2_-P and adsorbates (Sc(III) and Zr(IV)) was investigated by XPS. From the overall XPS spectrum of Figure 9a, it can be seen that the peaks of Sc 2p and Zr 3d appeared after the adsorption experiments of TRPO/SiO_2_-P, which indicates the successful adsorption of Sc(III) and Zr(IV) by the adsorbent. To further understand the adsorption mechanism, analysis of the high-resolution spectra of Sc, Zr, and P revealed that the peaks appearing at 399.72 or 399.06 eV in the narrow spectrum of Sc 2p (Figure 9b) were attributed to scandium metal [36], as the peaks of Sc 2p appeared at 402.84 eV, 407.21 eV, 402.60 eV, and 407.26 eV, which were very close to those of ScOOH (407.46 ± 0.08 eV and 402.97 ± 0.11 eV for Sc 2p1/2 and Sc 2p3/2, respectively) [50], suggesting that the adsorption of Sc(III) is bonded to the oxygen atoms on the surface of TRPO/SiO_2_-P. The two XPS peaks near 183 and 186 eV in Figure 9c are related to the oxidized valence state of Zr [51], and the binding energies for Zr 3d5/2 (183.15 and 182.82 eV) and Zr 3d3/2 (185.54 and 185.19 eV) are greater than those of Zr(OH)_4_ (182.2 eV); so, it can be determined that the adsorption of Zr(IV) was oxygen bonding with multiple P=O groups [52]. The ratio of the P-C peak area to the P=O peak area was found to be consistently around 3:1 in the narrow spectrum of P 2p (Figure 9d), which is in accordance with the theoretical value of TRPO [22]. Compared with the BE peaks of P-C and P=O in fresh TRPO/SiO_2_-P, the BE peaks of P-C and P=O of TRPO/SiO_2_-P were both shifted after the adsorption of scandium and zirconium in sulfuric and hydrochloric acid media, and it can be seen that a greater shift was produced in the adsorption of Zr(IV), which suggests that the adsorption process not only creates a new chemical bond but also the adsorbent has a higher affinity for Zr(IV).

### 3.3. Elution and Reusability of TRPO/SiO_2_-P

**Figure 10:**
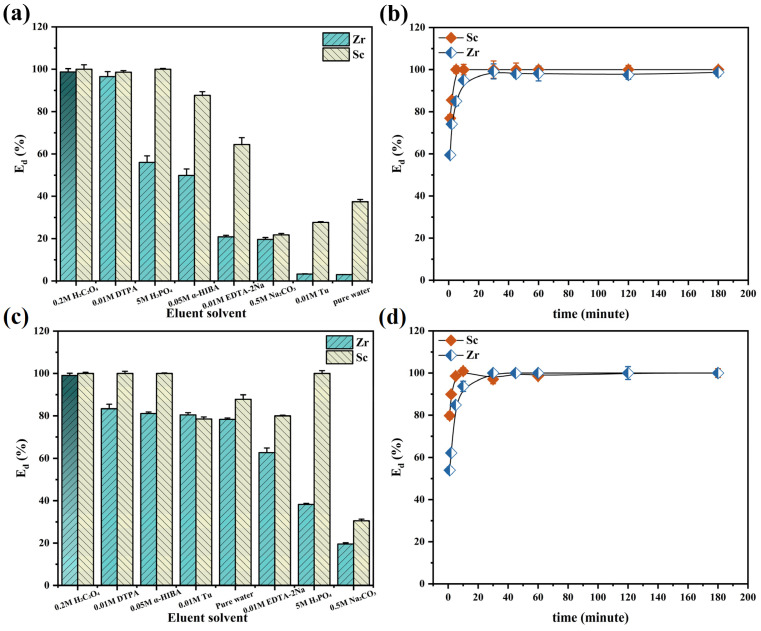
(**a**) Desorption properties and (**b**) desorption kinetics of Sc and Zr adsorbed in sulfuric acid medium, and (**c**) desorption properties and (**d**) desorption kinetics of Sc and Zr adsorbed in hydrochloric acid medium (adsorption conditions: m/V = 1 g/50 mL, (metal) = 8 mM, T = 298 K, shaking speed: 140 rpm, t = 24 h, medium: 0.2 M H_2_SO_4_ or 5 M HCl; desorption conditions: m/V = 0.1 g/5 mL, T = 298K, shaking speed: 140 rpm, eluent: 0.2 M H_2_C_2_O_4_).

The desorption properties of scandium and zirconium were assessed using eight different eluents to investigate the desorption kinetics. The results are presented in Figure 10. Notably, only 0.2 M H_2_C_2_O_4_ achieved a desorption efficiency exceeding 99% for Zr, making it a particularly suitable eluent given its cost-effectiveness. Figure 10b,d displays the desorption kinetics of scandium and zirconium using 0.2 M H_2_C_2_O_4_, which shows a rapid elution of both Sc and Zr, with nearly 100% elution accomplished within 30 min. This indicates that the eluent efficiently and swiftly removes scandium and zirconium ions adsorbed on the adsorbent’s surface.

**Figure 11:**
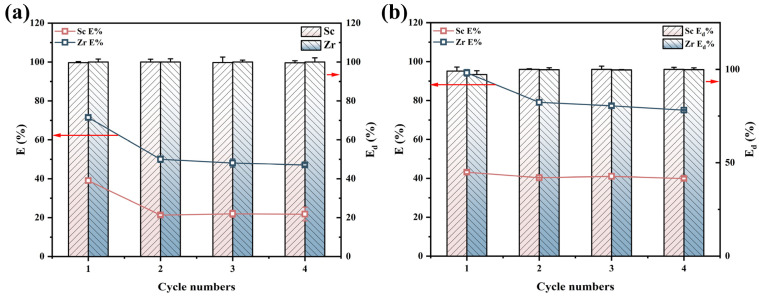
Regeneration of Sc and Zr by TRPO/SiO_2_-P in (**a**) 0.2 M H_2_SO_4_ and (**b**) 5 M HCl (adsorption conditions: m/V = 0.1 g/5 mL, (metal) = 8 mM, T = 298K, shaking speed: 140 rpm, t = 24 h; desorption conditions: m/V = 0.1 g/5 mL, T = 298K, shaking speed: 140 rpm, eluent: 0.2 M H_2_C_2_O_4_).

To assess the reusability of the adsorbent, four adsorption–desorption cycle experiments were conducted on TRPO/SiO_2_-P, with results depicted in Figure 11. TRPO/SiO_2_-P exhibited a decrease in adsorption efficiency after the initial cycle (18% and 21.5% for Sc and Zr in sulfuric acid medium, respectively (Figure 11a); while up to 3% and 15% for Sc and Zr in hydrochloric acid medium, respectively (Figure 11b)). However, efficiency remained stable in subsequent cycles. This is likely because H_2_C_2_O_4_ used in the initial desorption damaged some adsorption sites, rendering them unable to bind with Sc and Zr in subsequent adsorption processes. In summary, despite a significant performance loss following the initial adsorption–desorption cycle, TRPO/SiO_2_-P retains nearly 80% of its initial adsorption capacity after four cycles, indicating good reusability.

### 3.4. Column Separation Experiment

**Figure 12:**
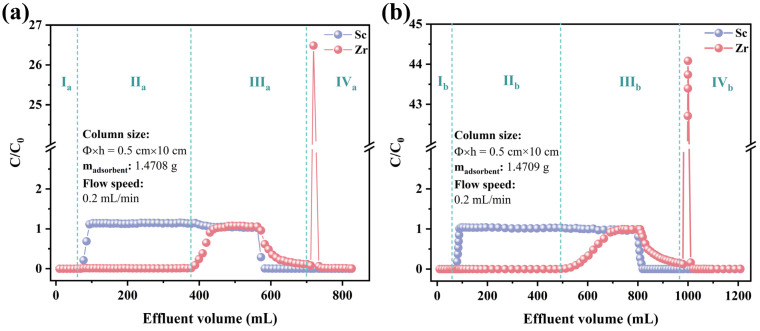
Chromatographic separation of Sc and Zr with TRPO/SiO_2_-P adsorbent packed columns in (**a**) 0.2 M H_2_SO_4_ and (**b**) 5 M HCl (I_a_: 0.2 M H_2_SO_4_, II_a_: Sc/Zr = 10 mM/1 mM mixed solution in 0.2 M H_2_SO_4_, III_a_: 0.2 M H_2_SO_4_, IV_a_: 0.2 M H_2_C_2_O_4_; I_b_: 5 M HCl, II_b_: Sc/Zr = 10 mM/1 mM mixed solution in 5 M HCl, III_b_: 5 M HCl, IV_b_: 0.2 M H_2_C_2_O_4_).

To simulate conditions for separating impurity of zirconium from scandium solution, a feed solution with a scandium–zirconium molar ratio of 10 was employed in the column experiments (Figure 12). The absence of ions in the effluent (C/C_0_ = 0) at the onset of section II_a_ in a sulfuric acid medium (Figure 12a) (C/C_0_ = 0) is attributed to the adsorbent surface possessing abundant adsorption sites, leading to the complete adsorption of Sc(III) and Zr(IV) that passed through the column. Following the initial penetration of Sc(III) from the column, its concentration in the tailing liquid gradually rose, reaching equilibrium at C/C_0_ = 1.13 ± 0.01. The C/C_0_ > 1 scenario is due to the depletion of adsorption sites, leading to the replacement of initially adsorbed Sc(III) with Zr(IV), which is attributed to the high affinity for Zr(IV). During this stage, almost no Zr(IV) was detected in the outset solution, resulting in a scandium purification rate of 99.8%. Similarly, in a hydrochloric acid medium (Figure 12b), the purification rate of Sc reached an even higher 99.99% in stage II_b_, so that the precise separation of scandium and zirconium could be realized in this stage. Stage IV encompasses the Zr elution process, wherein all Zr bound to the adsorbent is eluted using 0.2 M H_2_C_2_O_4_. Notably, this process does not involve Sc, allowing for effective recovery of Zr.

## 4. Conclusions

Static adsorption experiments conducted with TRPO/SiO_2_-P in sulfuric and hydrochloric acid media demonstrated outstanding scandium and zirconium separation, with separation factors approaching thousands. This suggests that TRPO/SiO_2_-P holds considerable potential as a candidate adsorbent for practical applications in scandium–zirconium separation scenarios. Fitting results from the combined pseudo-secondary and Weber–Morris models in kinetic experiments suggest that TRPO/SiO_2_-P’s adsorption of scandium and zirconium is primarily governed by chemisorption, while also being influenced by internal diffusion processes. Isotherm data align more closely with the Langmuir model, denoting that the adsorption process entails homogeneous monolayer adsorption. Thermodynamic analysis results reveal that TRPO/SiO_2_-P’s adsorption of zirconium ions is both spontaneous and endothermic.

The economically viable H_2_C_2_O_4_, employed in elution experiments, serves as an effective eluent for scandium and zirconium, exhibiting rapid desorption kinetics and nearly 100% elution efficiency. Furthermore, TRPO/SiO_2_-P demonstrates good reusability, maintaining approximately 80% of its original adsorption performance even after four adsorption–desorption cycles. SEM-EDS, FT-IR, and XPS results revealed that TRPO/SiO_2_-P uniformly adsorbed scandium and zirconium on both the surface and inner surface of the resin. The involvement of SO_4_^2−^ and Cl^−^ throughout the adsorption process, and the differing affinities of the P=O groups for scandium and zirconium, were pivotal in achieving scandium–zirconium separation. Column separation experiments demonstrated that TRPO/SiO_2_-P can produce scandium solutions with purities of 99.8% in sulfuric acid and 99.99% in hydrochloric acid, respectively, confirming the practical applicability of TRPO/SiO_2_-P.

## Figures and Tables

**Table 1:** Comparison of separation performance of three resins in sulfuric and hydrochloric acid media.

Resin	Acid	*SF*_*Zr*/*Sc*_	Source
TRPO/SiO_2_-P	0.2 M H_2_SO_4_	380	Figure 2a
	5 M HCl	977	Figure 2d
TRPO/XAD7HP	0.5 M H_2_SO_4_	178	Figure 2b
	4 M HCl	91	Figure 2e
TRPO/HZ-635	0.2 M H_2_SO_4_	239	Figure 2c
	5 M HCl	297	Figure 2f

**Table 2:** Kinetic fitting parameters for Sc(III) and Zr(IV) in 0.2 M H_2_SO_4_ solution.

Elements	T(K)	Pseudo-First-Order			Pseudo-Second-Order			$Q_{e,exp}$ (mg·g^−1^)
		*K*_1_ (min^−1^)	$Q_{e}$ (mg·g^−1^)	*R* ^2^	*K*_2_ (g·mg^−1^·min^−1^)	$Q_{e}$ (mg·g^−1^)	*R* ^2^	
Sc	298	0.86	7.00	0.94	0.21	7.23	0.99	7.18
Zr	298	0.39	29.48	0.81	0.02	30.38	0.99	30.5
	308	0.44	29.99	0.83	0.03	30.83	0.99	30.9
	318	0.67	30.14	0.85	0.05	30.79	0.99	30.4

**Table 3:** Kinetic fitting parameters for Sc(III) and Zr(IV) in 5 M HCl solution.

Elements	T(K)	Pseudo-First-Order			Pseudo-Second-Order			$Q_{e,exp}$ (mg·g^−1^)
		*K*_1_ (min^−1^)	*Q*_e_ (mg·g^−1^)	*R* ^2^	*K*_2_ (g·mg^−1^·min^−1^)	*Q*_e_ (mg·g^−1^)	*R* ^2^	
Sc	298	0.31	7.20	0.87	0.07	7.42	0.99	7.41
Zr	298	0.09	34.60	0.86	0.004	36.64	0.98	37.11
	308	0.13	36.01	0.85	0.006	37.64	0.98	38.80
	318	0.17	38.52	0.93	0.007	39.87	1	39.62

**Table 4:** Isotherm fitting parameters for Sc(III) and Zr(IV) in 0.2 M H_2_SO_4_ solution.

Elements	T(K)	Langmuir			Freundlich			Redlich–Peterson				$Q_{e,exp}$ (mg·g^−1^)
		*K_L_* (L·mg^−1^)	$q_{m}$ (mg·g^−1^)	*R* ^2^	*K*_F_ (L·g^−1^)	*n*	*R* ^2^	*A*	*B*	*g*	*R* ^2^	
Sc	298	0.01	10.71	0.996	0.74	2.34	0.964	0.11	0.001	1.01	0.996	8.00
Zr	298	0.06	32.83	0.985	9.52	4.67	0.797	1.54	0.026	1.10	0.998	30.26
	308	0.08	32.76	0.975	10.43	4.99	0.783	1.98	0.035	1.10	0.987	30.98
	318	0.13	32.10	0.941	11.94	5.64	0.772	3.42	0.080	1.05	0.941	31.28

**Table 5:** Isotherm fitting parameters for Sc(III) and Zr(IV) in 5 M HCl solution.

Elements	T (K)	Langmuir			Freundlich			Redlich–Peterson				$Q_{e,exp}$ (mg·g^−1^)
		*K_L_* (L·mg^−1^)	$q_{m}$ (mg·g^−1^)	*R* ^2^	*K*_F_ (L·g^−1^)	*n*	*R* ^2^	*A*	*B*	*g*	*R* ^2^	
Sc	298	0.04	8.77	0.976	2.10	4.04	0.965	0.65	0.15	0.87	0.995	8.31
Zr	298	1.25	38.03	0.987	20.59	6.98	0.754	47.86	1.27	1	0.985	38.65
	308	1.53	38.82	0.987	21.62	7.13	0.743	58.77	1.5	1	0.987	39.54
	318	1.71	39.66	0.986	22.41	7.03	0.740	67.34	1.69	1	0.984	40.12

**Table 6:** Thermodynamic fitting parameters of TRPO/SiO_2_−P to Zr(IV) in different media.

Media	${∆H}^{0}$ (kJ/mol)	${∆S}^{0}$ (kJ/K·mol)	${∆G}^{0}$ (kJ/mol)			$R^{2}$
			298 K	308 K	318 K	
0.2 M H_2_SO_4_	28.19	0.20	−22.64	−24.84	−35.41	0.92
5 M HCl	12.35	0.14	−29.37	−30.77	−32.17	0.95

## Data Availability

Data are contained within the article and Supplementary Materials.

## Supplementary Materials

The following supporting information can be downloaded at: https://www.mdpi.com/article/10.3390/toxics12050350/s1. Figure S1. Chemical structures of (a) XAD7HP and (b) HZ-635 (R means Alkyl). Figure S2. Simple schematic diagram of the column system. Figure S3. Effect of contact time on the adsorption performance (m/V = 0.1 g/5 mL, Sc(III)/Zr(IV) = 5 mM/5 mM, T = 298 K, shaking speed: 140 rpm, medium: (a) in 0.2 M H2SO4, (b) in 5 M HCl solutions). Table S1 Internal diffusion model fitting data in 0.2 M H2SO4 solution. Table S2 Internal diffusion model fitting data in 5 M HCl solution. References [38,43,53,54,55,56] are cited in the Supplementary Materials.
