# Supplementary material for: Study of the Adsorption and Separation Behavior of Scandium and Zirconium by Trialkyl Phosphine Oxide-Modified Resins in Sulfuric and Hydrochloric Acid Media
Source: Toxics. 2024 May 10;12(5):350. doi: 10.3390/toxics12050350 (PMC11125866; doi:10.3390/toxics12050350)
Supplement: Supplementary file 1 [file toxics-12-00350-s001.zip › toxics-2991465-supplementary.pdf]

Supporting Information (SI) for

Study of the adsorption and separation behavior of scandium and zirconium by trialkyl phosphine oxide-modified resins in sulfuric and hydrochloric acid media

Botao Xu [a], Xiangbiao Yin *[,b], Shunyan Ning [b], Yilai Zhong [a], Toyohisa Fujita [a], Mohammed F. Hamza [b], Yuezhou Wei [b, c,]*

[a] *State Key Laboratory of Featured Metal Materials and Life-cycle Safety for Composite Structures, MOE Key Laboratory of New Processing Technology for Nonferrous Metals and Materials, and School of Resources, Environment and Materials, Guangxi University, Nanning 530004, P.R. China,*

[b] *School of Nuclear Science and Technology, University of South China, 28 Changsheng West Road, Hengyang 421001, P.R. China.*

[c] *School of Nuclear Science and Engineering, Shanghai Jiao Tong University, 800 Dong Chuan Road, Shanghai 200240, China*

* Address: *School of Nuclear Science and Technology, University of South China, 28 Changsheng West Road, Hengyang 421001, PR China*

Email: yinxb@usc.edu.cn

ORCID: 0000-0003-2319-1789

* Address: *School of Nuclear Science and Technology, University of South China, 28 Changsheng West Road, Hengyang 421001, PR China*

Email: yzwei@usc.edu.cn

ORCID: 0000-0003-3821-9078

Toxics

*To Whom Correspondence Should Be Addressed

Contents:

5 Pages (Including cover page)

2 tables

3 figures

2 Experimental

2.1. Materials

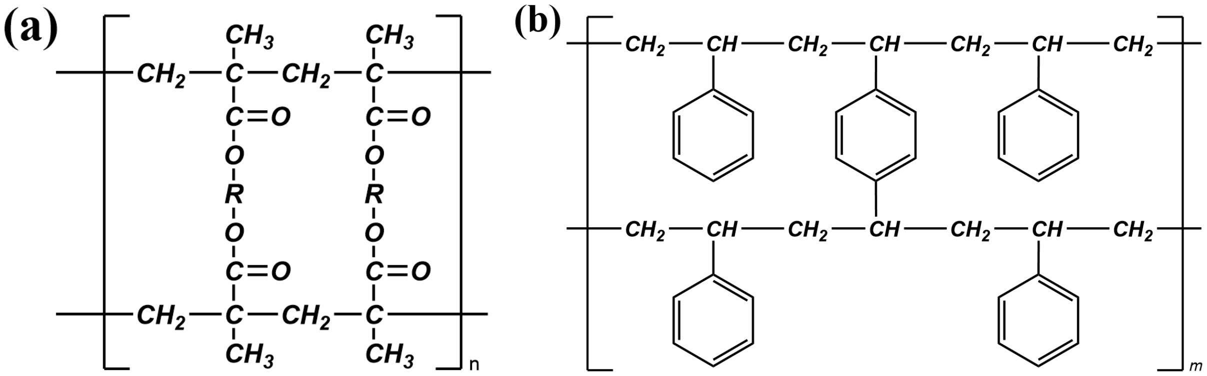

Fig. S1. Chemical structures of (a) XAD7HP and (b) HZ-635 (*R* means Alkyl)

2.5. Column separation experiment

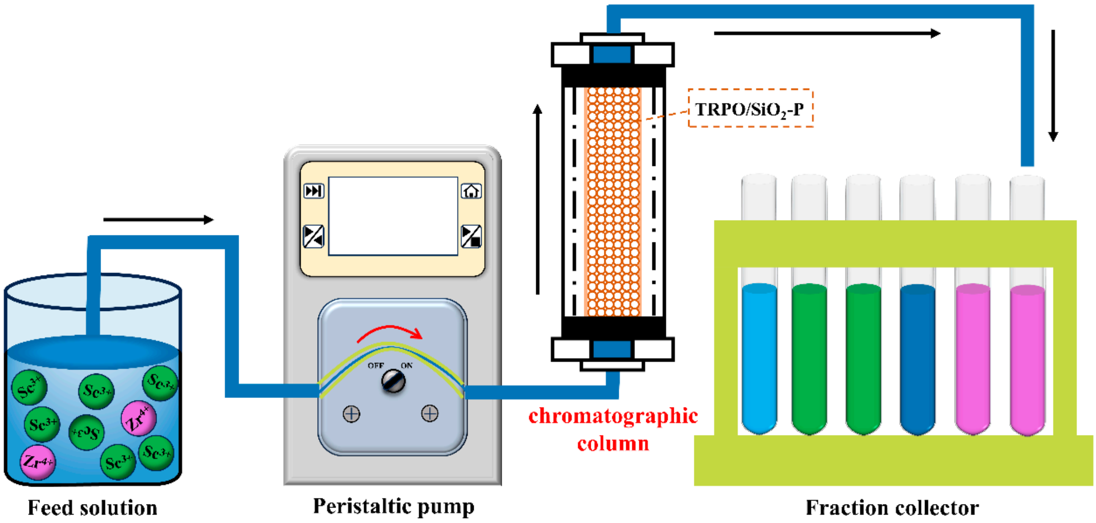

Fig. S2. Simple schematic diagram of the column system

3. Results and discussion

3.1.1. Effect of carrier and acidity

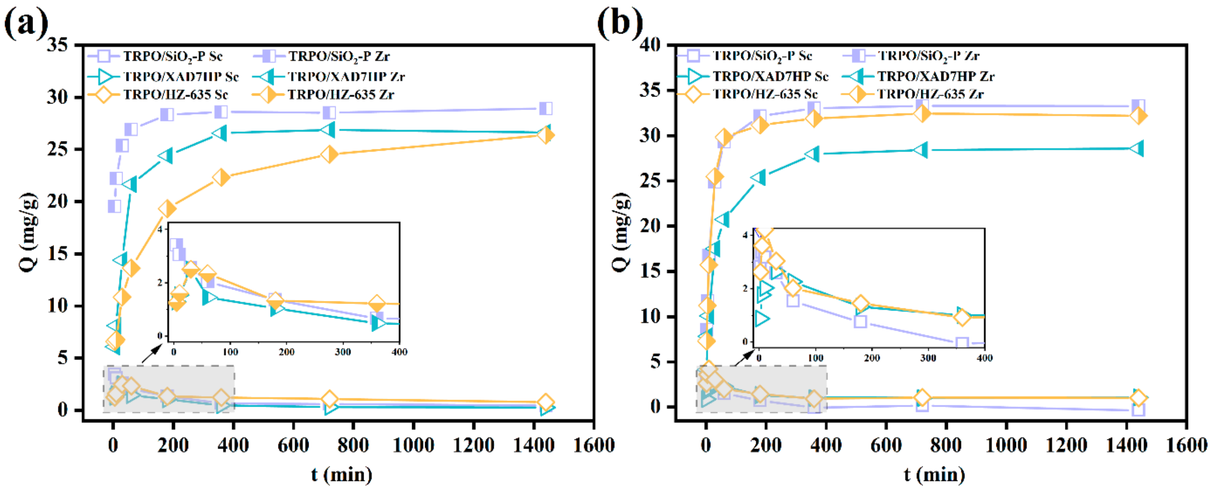

Fig. S3. Effect of contact time on the adsorption performance (m/V = 0.1 g/5 mL, Sc(III)/Zr(IV) = 5 mM/5 mM, T = 298 K, shaking speed : 140 rpm, medium: (a) in 0.2 M H_2SO_4, (b) in 5 M HCl solutions)

3.1.4. Kinetic analysis

In this work, both pseudo-first-order kinetics model (Eq. S1), pseudo-second-order kinetic model (Eq. S2), and Weber-Morris internal diffusion model (Eq. S3) were applied to analyze the experimental data[1, 2].

$$\ln(Q_e - Q_t) = \ln(Q_e) - k_1 t \qquad \text{(S1)}$$

$$\frac{t}{Q_t} = \left(\frac{1}{Q_e}\right) t + \frac{1}{k_2 {Q_e}^2} \qquad \text{(S2)}$$

$$Q_t = k_p t^{1/2} + C \qquad \text{(S3)}$$

Where Q_e ($mg \cdot g^{-1}$) and Q_t ($mg \cdot g^{-1}$) are the equilibrium adsorption capacity and adsorption capacity at time t (h) respectively; k_1 (h^{-1}), k_2 ($mg \cdot g^{-1}\ h^{-1}$) and kp ($mg \cdot g^{-1} \cdot h^{-1/2}$) are the adsorption rate constant of pseudo-first-order, pseudo-second-order and intra-particle diffusion model respectively. C is a constant associated with the thickness of the boundary layer.

Table S1 Internal diffusion model fitting data in 0.2 M H_2SO_4 solution.

Element	T (K)	Step 1			Step 2			Step 3		
		K_{P1} ($mg \cdot g^{-1} \cdot h^{-1/2}$)	C_1	R_1^2	K_{P2}	C_2	R_2^2	K_{P3}	C_3	R_3^2
Sc	298									

Zr	298	3.28	68.1	0.98	1.12	75.30	0.99	0.04	87.16	0.80
	308	4.09	68.2	0.99	1.07	78.13	0.99	0.05	89.02	0.96
	310	5.53	67.99	0.98	0.88	82.98	1	0.05	92.09	0.92

Table S2 Internal diffusion model fitting data in 5 M HCl solution.

Element	T (K)	Step 1			Step 2			Step 3		
		K_{P1} (mg·g^{-1}·h$^{-1/2}$)	C_1	R_1^2	K_{P2}	C_2	R_2^2	K_{P3}	C_3	R_3^2
Sc	298									
Zr	298	3.28	68.1	0.98	1.12	75.30	0.99	0.04	87.16	0.80
	308	4.09	68.2	0.99	1.07	78.13	0.99	0.05	89.02	0.96
	310	5.53	67.99	0.98	0.88	82.98	1	0.05	92.09	0.92

3.1.5. Adsorption isothermal

The isothermal adsorption of TRPO/SiO_2-P was analyzed by Langmuir equation (Eq. S4), Freundlich equation (Eq. S5) and Redlich-Peterson equation (Eq. S6)[3-6].

$$Q_e = \frac{q_m \times K_L \times C_e}{1 + K_L \times C_e} \tag{S4}$$

$$Q_e = K_F \times C_e^{\frac{1}{n}} \tag{S5}$$

$$Q_e = \frac{A \times C_e}{1 + B \times C_e^g} \tag{S6}$$

where Q_e (mg·g^{-1}) and q_m (mg·g^{-1}) are equilibrium adsorption capacity and calculated saturation adsorption capacity; C_e (mmol·L^{-1}) means equilibrium ions concentration; K_L (L·mg^{-1}) and K_F (mg^{1-n}·L^n/g) are constants of Langmuir and Freundlich isotherm model; n means adsorption intensity; where A, B, and g are the Redliche-Peterson parameters.

References

[1] W. Ma, T.T. Lv, J.H. Tang, M.L. Feng, X.Y. Huang, Highly Efficient Uptake of Cs^{+} by Robust Layered Metal-Organic Frameworks with a Distinctive Ion Exchange Mechanism, JACS Au, 2 (2022) 492-501.

[2] S. Zhang, S. Ning, H. Liu, X. Wang, Y. Wei, X. Yin, Preparation of ion-exchange resin via in-situ polymerization for highly selective separation and continuous removal of palladium from electroplating wastewater, Separation and Purification Technology, 258 (2021).

[3] M. Vigdorowitsch, A. Pchelintsev, L. Tsygankova, E. Tanygina, Freundlich Isotherm: An Adsorption Model Complete Framework, Applied Sciences, 11 (2021).

[4] M.A. Al-Ghouti, D.A. Da'ana, Guidelines for the use and interpretation of adsorption isotherm models: A review, J Hazard Mater, 393 (2020) 122383.

[5] K.Y. Foo, B.H. Hameed, Insights into the modeling of adsorption isotherm systems, Chemical Engineering Journal, 156 (2010) 2-10.

[6] R. Han, W. Zou, Y. Wang, L. Zhu, Removal of uranium(VI) from aqueous solutions by manganese oxide coated zeolite: discussion of adsorption isotherms and pH effect, J Environ Radioact, 93 (2007) 127-143.
